# Supplementary material for: "I’m going to stay young": Belief in anti-aging efficacy of menopausal hormone therapy drives prolonged use despite medical risks
Source: PLoS One. 2020 May 29;15(5):e0233703. doi: 10.1371/journal.pone.0233703 (PMC7259599; doi:10.1371/journal.pone.0233703)
Supplement: Attachment — Submitted filename: Reviewer comments.docx [file pone.0233703.s001.docx]

Dear editor of Plos One Journal

Greetings

Thank you very much for sending this manuscript for review

The following are suggested:

•	The topic is well-introduced and the problem stated

•	The methods and results are fair. The authors are kindly invited to provide the reference number and date of the ethical clearance letter

•	Some of the references need revision (ref.14, and 20)

I strongly recommend the manuscript for publication

Best regards

Dr. Hyder Mirghani, MD, MSc, Associate Prof. of Clinical Medicine, And Endocrine, Department of Internal Medicine, Medical College, University of Tabuk
